# Short-Term Outcomes and Efficacy of Percutaneous Deep Vein Arterialization for No-Option Critical Limb Ischemia: A Systematic Review and Meta-Analysis

**DOI:** 10.3390/biomedicines12020318

**Published:** 2024-01-30

**Authors:** Roshanak Roustazadeh, Alexander Gombert, Julia Krabbe, Michael Jacobs, Panagiotis Doukas

**Affiliations:** 1 European Vascular Centre Aachen-Maastricht, Department of Vascular Surgery, University Hospital RWTH Aachen, 52074 Aachen, Germany; agombert@ukaachen.de (A.G.); m.jacobs@mumc.nl (M.J.); pdoukas@ukaachen.de (P.D.); 2 Institute of Occupational, Social and Environmental Medicine, Medical Faculty, RWTH Aachen University, Pauwelsstraße 30, 52074 Aachen, Germany

**Keywords:** chronic limb-threatening ischemia, meta-analysis, percutaneous deep vein arterialization, systematic review

## Abstract

Background: Percutaneous deep vein arterialization (pDVA) is considered a treatment modality in patients with no-option critical limb ischemia. However, there is still a paucity of evidence regarding its safety and efficacy. Data sources: MEDLINE (via PubMed), Embase and Web of Science databases as well as the CENTRAL registry up to the end of June 2023. Methods: This review adhered to the PRISMA guidelines (PROSPERO registration no. CRD42023445171). The risk of bias was assessed using the methodological index for non-randomized studies (MINORS). Primary endpoints included technical success, overall survival and limb salvage during the follow-up. Amputation-free survival at 30 days, 6 months and 1 year as well as complete wound healing, major adverse limb events and reintervention were investigated as secondary outcomes. Results: Five observational studies, comprising 208 patients (142 Rutherford class 5/77 Rutherford class 6), were included. MINORS revealed a low risk of bias. The meta-analysis reached a pooled technical success rate of 96.2% (95% CI: 91.5–98.4), an overall survival of 82.8% (95% CI: 70.5–95.2) and a limb salvage rate of 77.2% (95% CI: 65.2–89.1) during the follow-up. The amputation-free survival at 30 days, 6 months and 1 year was 87.8%, 68.7% and 65.6%, respectively. Furthermore, pDVA resulted in a complete wound healing rate of 53.4% (95% CI: 30.3–76.5). The pooled reintervention rate was as high as 46.7% (37.1–56.3%). Conclusions: PDVA seems a feasible bail-out strategy for patients with no option for routine treatment of CLTI. However, due to the small number of studies, the strength of the evidence is low.

## 1. Introduction

The advanced stage of peripheral arterial disease (PAD), known as chronic limb-threatening ischemia (CLTI), afflicts approximately 11% of PAD patients annually [1]. This condition is linked to elevated mortality and amputation rates, reaching as high as 25% and 30% within the first year, respectively [2], and is also associated with impaired quality of life [3,4]. Half of the individuals subjected to below-knee amputations remain immobilized one year following the procedure and 21% do not survive the initial 6 months [5]. Endovascular interventions, open surgery and hybrid procedures are opted for to prevent amputation and promote wound healing and/or resolve rest pain [3]. Nevertheless, despite advancements in these treatment modalities, a minimum of 20% of CLTI patients are deemed unsuitable for arterial reconstruction [6]. In these cases, further referred to as no-option CLTI, both endovascular and surgical procedures are unfeasible or have failed mainly due to the absence of a suitable distal run-off vessel [7].

In order to provide arterial perfusion to the foot’s capillary bed in cases of no-option CLTI, the concept of venous arterialization has emerged as a viable alternative. Originally introduced by Hastead and Vaughan in 1912 [8], this concept has undergone continuous refinement and has evolved into completely percutaneous techniques in the 21st century. In 2017, Kum et al. presented a case series involving seven no-option CLTI patients who successfully underwent percutaneous deep vein arterialization (pDVA) and reported a 71% limb salvage rate at the 12-month mark [9]. The core principle of pDVA involves establishing an arteriovenous fistula (AVF) proximal to the diseased segment of the tibial arteries. The requirements for pDVA are at least one patent proximal tibial artery as the inflow and patent plantar and/or dorsal deep vein arch(es) as the outflow [10]. The procedure may be performed using either a specialized device (e.g., LimFlow, SA, Paris, France) or conventionally using available re-entry tools to create the AVF. Subsequently, the guidewire is advanced through the target foot vein and the valvulotomy is performed using balloon dilatation, cutting balloons or a valvulotome. The deep vein arterialization may be proceeded by deploying a self-expandable stent graft in the tibial vein and a tapered one across the arteriovenous crossing point [10,11]. This redirection of blood flow diverts it from the tibial artery to the deep vein through the use of a covered stent graft, which prevents leakage at the arteriovenous connection and restricts blood flow from entering the proximal vein segment, thus avoiding venous run-off towards the heart.

Despite recent studies demonstrating promising outcomes for pDVA in the treatment of patients with no-option CLTI and a meta-analysis on the effectiveness of all surgical and endovascular approaches for venous arterialization reporting a notable 79% 1-year limb salvage rate [12], the evidence level remains low and this procedure has not yet secured a definitive position in routine clinical practice. The primary objective of the present systematic review is to investigate the technical feasibility of pDVA and assess the outcomes of patients with no-option CLTI following the procedure.

## 2. Materials and Methods

This systematic review and meta-analysis was designed and conducted according to the Preferred Reporting Items for Systematic Reviews and Meta-Analyses (PRISMA) statement [13] and is registered with PROSPERO, CRD42023445171.

### 2.1. Search Strategy and Eligibility Criteria

We performed a systematic search for relevant studies in the MEDLINE (via PubMed), Embase and Web of Science databases as well as the CENTRAL registry using the search strategy outlined in the Supplementary Materials (Supplementary Table S2). Bibliographies of the included studies, citations and review articles were manually scrutinized as a complementary source. Studies in languages other than English and grey literature were not sought. This yielded a battery of studies published up to the end of June 2023 on the efficacy and adverse events of percutaneous deep venous arterialization in patients with no-option critical limb-threatening ischemia.

Two reviewers (R. R. and P. D.) independently used a priori PICOS (population, intervention, comparator, outcome and study design) to choose published papers that were pertinent to the goals of the current meta-analysis. The population of interest comprised of patients with no-option CLTI, who had previously not responded to any revascularization procedures and had no viable options for further arterial reconstruction surgery. However, they still had a patent below-the-knee artery that could be used as an inflow vessel for an arteriovenous fistula. Only patients with distal foot ulcerations and tissue necrosis (Rutherford category 5 or 6) were included. Percutaneous deep vein arterialization (pDVA) was defined as the intervention of interest. Moreover, the search was not restricted to studies performing pDVA using any particular device. No particular comparator was defined. Excluded were case reports, studies involving less than 15 patients of interest, letters, editorials and reviews. We took the information from the most recent study with the biggest sample size for the pertinent outcome when the same cohort was reported twice.

Primary endpoints were the technical success rate (defined as the ability to cross from the artery in the vein and successful implantation of the stent graft with detectable flow within) [14], limb salvage during the follow-up (defined as the absence of an ipsilateral above-ankle amputation) and overall survival during the follow-up.

Limb salvage, overall survival and their composite, amputation-free survival, were also assessed at 30 days, 6 months and 1 year as secondary endpoints. Further secondary endpoints included reintervention rate, procedural success rate (the combination of technical success, amputation-free survival and freedom from reintervention within 30-days after the procedure), patency rates, major adverse limb events (MALEs), major adverse cardiovascular events (MACEs), change in Rutherford classification and complete wound healing. Patency was defined as primary if no intervention or stenosis was reported during the follow-up period [15], as primary assisted if reintervention was necessary for restenosis in the absence of occlusion [10] and secondary if reintervention was required to restore patency [15]. MALEs were defined as a loss of patency of the revascularization, reintervention on the revascularized segment or major amputation (above-the-ankle) of the revascularized limb [16]. MACEs were defined as cerebrovascular incident, myocardial infarction or death during the follow-up [16].

### 2.2. Data Extraction and Quality Assessment

Two researchers independently (R. R. and P. D.) extracted the data from each study and the final decision was reached by consensus. Collected data included publication data (study name, publication year, country), study design, follow-up period, baseline characteristics of the participants (number of patients, age, sex, number with Rutherford classification 5 or 6, smoking, diabetes mellitus, hypertension, chronic kidney disease, dyslipidemia, drug history, antiplatelet administration and preprocedural WIfI score (Wound, Ischemia, foot Infection)) and outcome data.

The validity and risk of bias of the included studies were assessed by two independent reviewers (R. R. and P. D.) using the (methodological index for non-randomized studies) MINORS criteria [17]. Inconsistencies were resolved by seeking the opinion of a third reviewer (A. G.).

### 2.3. Statistical Analysis

The Comprehensive Meta-Analysis software package version 3.0 (Biostat, Englewood, NJ, USA) was used to analyze all the datasets. To pool the data, the mean ± SD for continuous variables and the proportion of patients for dichotomous variables were gathered, and the heterogeneity of the studies was assessed using the Chi-squared-based Q-statistic test [18]. If studies reported either medians and ranges or individual patient data, the desired effects of the measures were calculated using these data. If the *p*-value of the heterogeneity Q-statistic did not reduce upon the sensitivity analysis, the random effects model was employed to compute the pooled effect sizes. The fixed-effects model was implemented otherwise. The impacts of heterogeneity were quantified using I^2^ statistics. I^2^ values of 25, 50 and 75%, respectively, denoted minimal, moderate and high heterogeneity. Sensitivity analysis was undertaken to investigate sources of heterogeneity. Due to the low number of included studies in the meta-analysis, subgroup analysis was not feasible. Individual studies were excluded sequentially in terms of the sensitivity analyses to evaluate the robustness of the results. Significant changes (>20%) observed in the outcome after the removal of each study led to its exclusion to avoid selection bias before the analysis was rerun. Univariate meta-regression was performed to explore the association of outcomes and baseline patients and procedural characteristics. Egger’s test was employed to detect the publication bias, which was then treated using Duval and Tweedie’s trim-and-fill method [19,20,21]. The results were considered significant at *p* < 0.05.

## 3. Results

### 3.1. Study Design

**Figure 1:**
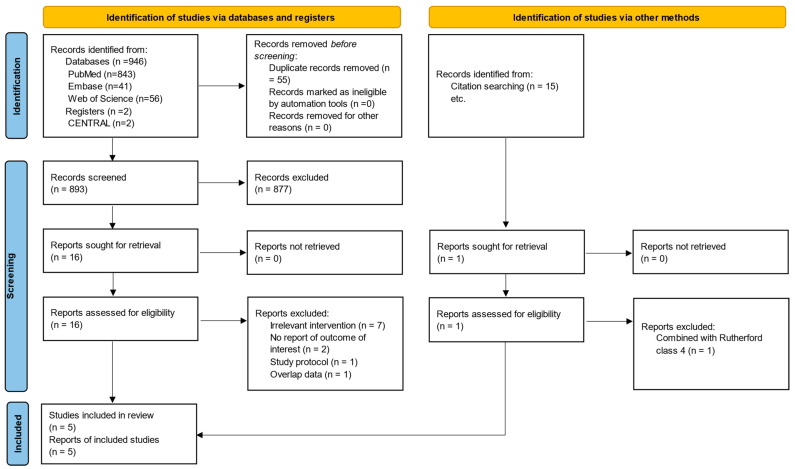
PRISMA flow diagram.

A rigorous literature search yielded 963 records, from which 5 studies ultimately met the inclusion criteria (3 prospective and 2 retrospective observational studies) [10,11,14,15,22]. The PRISMA flow diagram (Figure 1) comprehensively depicts the systematic search and the exclusion process at each stage. The characteristics of the included studies are presented in Table 1. The complete search strategy is displayed in Supplementary Table S1 and Table S2 provides detailed information on the excluded studies upon full-text scrutiny, including the reasons for their exclusion [23,24,25,26,27,28,29,30,31,32,33,34].

### 3.2. Study Population

A total of 208 patients were included in the meta-analysis. The mean age was 70.02 years (95% CI: 67.55–72.49) and 68.75% were men. The weighted mean follow-up was 13.44 (95% CI: 9.13–17.75) months. The median sample size was 32 patients (18–105), 156 patients (75%) were diagnosed with diabetes and 97 (46.64%) had chronic kidney disease. Table 2 summarizes the baseline characteristics of the treated patients.

### 3.3. Risk of Bias Assessment

According to the MINORS criteria the five included studies scored a mean of 12 (range 11–13) out of 16 points, indicating a low risk of bias. The quality criterium not met by any of the studies was the unbiased assessment of the study endpoint. The detailed risk of bias assessment is displayed in Table 3.

### 3.4. Outcomes

**Figure 2:**
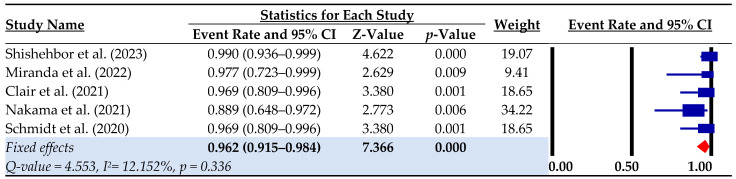
Technical success rate. Event rate = rate of successful pDVA; CI = confidence interval [10,11,14,15,22].

The technical success rate across the included studies ranged from 0.889 to 0.99, with a pooled estimate of 0.962 (95% CI: 0.915–0.984; I^2^ = 0.00%) (Figure 2). The pooled procedural success rate was 0.74 (95% CI: 0.665–0.803; I^2^ = 0.00%) (Table 4).

**Figure 3:**
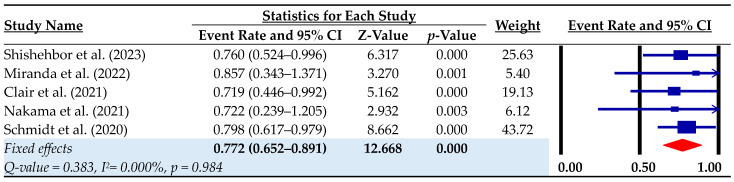
Limb salvage rate during the follow-up. Event rate = rate of successful pDVA; CI = confidence interval [10,11,14,15,22].

Limb salvage during the follow-up was achieved in 0.772 (95% CI: 0.652–0.891; I^2^ = 0.00%) of patients in the pooled analysis (Figure 3). Limb salvage rates at 30 days, 6 months and 1 year were 0.904, 0.771 and 0.767, respectively. Major adverse limb events (MALEs) occurred in 0.537 of patients, and complete wound healing was achieved in 0.534 of cases (Table 4). However, the heterogeneity between studies for the wound healing outcome was high (I^2^ = 78.53%, *p* = 0.001), suggesting the need for further investigation. The pooled reintervention rate during follow-up was 0.467 (95% CI: 0.371–0.563; I^2^ = 45.95%, *p* = 0.136), indicating that approximately one in two patients required further intervention.

**Figure 4:**
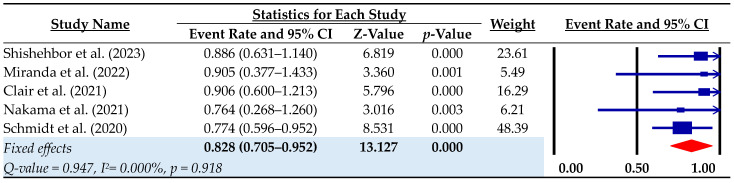
Overall survival rate during the follow-up. Event rate = rate of successful pDVA; CI = confidence interval [10,11,14,15,22].

Overall survival during follow-up was 0.828 (95% CI: 0.705–0.952; I^2^ = 0.00%), with a range of 0.764 to 0.906 across studies (Figure 4). Pooled survival rates at 30 days, 6 months and 1 year were 0.961, 0.90 and 0.851, respectively (Table 4). The pooled rates for the composite outcome of amputation-free survival were 0.878 at 30 days, 0.687 at 6 months and 0.656 at 1 year (Table 4).

Univariate meta-regression analysis revealed that diabetes mellitus was moderately inversely correlated with complete wound healing (R^2^ = 0.41; co-efficient= −1.63; goodness of fit *p* = 0.041, I^2^ = 63.46%). There was no other significant association between comorbidities and overall survival or limb salvage. Egger’s test for small-size studies detected no publication bias (*p* < 0.05).

## 4. Discussion

Advanced below-the-knee arterial disease leaves approximately 20% of patients with chronic limb-threatening ischemia not amenable to conventional well-established open and endovascular revascularization techniques such that an amputation often becomes inevitable [3,35,36]. Most of these patients are considered not suitable for arterial revascularization as they lack an outflow in the foot due to small-artery disease [37]. In coherence with this and as presented in Table 2, the vast majority of the patients who underwent pDVA in the current systematic review suffer from the risk factors for microvascular disease, such as diabetes mellitus and chronic kidney disease [38]. Although open deep vein arterialization was first introduced about 100 years ago, it was never undertaken as an established treatment strategy in CLTI patients [38]. However, with the recent advances in endovascular strategies and the introduction of off-the-shelf devices, percutaneous DVA could be a promising alternative for such patients [38]. Ucci et al. (2023) recently conducted a meta-analysis and investigated venous arterialization in CLTI patients [39]. However, they focused only on efficacy outcomes, leaving the associated safety issues and reintervention rates undiscussed; they also did not report on early perioperative (30 days) outcomes [39]. A further potential limitation of previous systematic reviews was the inclusion of case reports and case series involving less than 15 participants [12,39], running an increased risk of bias [40].

Our results showed that pDVA is a feasible technique with a technical success rate of 96.2%. The overall survival in the present analysis decreased slightly from 96.1% at 30 days postoperatively to 85.1% at 1 year. In comparison to Ucci et al. (2023), the overall survival rate at 6 months and 1 year in our analysis was slightly lower (92.8% vs. 90.0% and 87.2% vs. 85.1%, respectively) [39].

The limb salvage rates at 30 days, 6 months and 1 year in the current review were 90.4%, 77.1% and 76.7%, respectively, indicating that major amputations mostly tend to occur during the first 6 months after the procedure. In agreement with our findings, the PROMISE I and II studies reported comparable declines in limb salvage rates and AFS during the first 6 months of the follow-up, only to reach a relative plateau after up to 1 year, demonstrating that the first 6 months are the most critical period regarding limb salvage [10,22]. The pooled 1-year limb salvage rate in our analysis was slightly lower (76.7% vs. 78.6%) than that reported by Ucci et al. (2023) [39]. A meta-analysis by Ghare et al. (2021) reported AFS rate of 58.6% and 50.3% at 6 months and 1 year, respectively, in patients with no-option CLTI undergoing standard care conservative therapy [41]. A comparison of this finding with those of the present study indicates that pDVA leads to higher AFS rates (68.7% and 65.6%, respectively) at 6 months and 1 year in no-option CLTI patients.

According to the current analysis, 53.4% of the target wounds achieved complete healing during a pooled follow-up of 13 months in patients receiving pDVA. This is, however, significantly lower than that reported by Ucci et al. (2023) at 12 months (53.4% vs. 64.5%) [39]. The higher wound healing rate reported in the previous study arises from the higher wound healing estimates reported in the small case series included in the preset analysis [9,39,42]. However, it is important to note that this outcome is subject to large heterogeneity (I^2^ = 78.53%, *p*-value = 0.001), which may be rooted in the different follow-up durations among the included studies.

Moreover, approximately half of the patients receiving pDVA (46.7%) needed one or multiple reinterventions. This important outcome has not been thoroughly investigated in previous meta-analyses. It is important to note that DVA circuit occlusion was the main reason for reinterventions, followed by a need to optimize the arterial inflow of the fistula due to newly developed ulcers [10,14,15,22]. Only two of the included studies reported patency rates, which were pooled to a primary patency rate of 30.8% (95% CI: 21.8–39.7%). No sufficient data were available to estimate the primary assisted and secondary patency rates. However, Shishehbor et al. (2023) reported satisfactory primary assisted and secondary patency rates as high as 45.4% and 64.2%, respectively [10]. This highlights the importance of close surveillance to maintain the patency of the DVA circuit up to complete healing of the active wounds.

The pooled estimate of MALEs over the follow-up period was 53.7% (95% CI: 37.3–70.0%). Given that MALEs are defined as the composite outcome of both reinterventions and major amputations and considering the calculated limb salvage and reintervention rates measured in the current study, one might conclude that the reported high MALE rate is mostly due to the high rate of reinterventions rather than that of amputations. However, scant data were found on the safety outcomes and complications of pDVA, with only three studies reporting MALEs during the follow-up [10,11,15]. Although Yan et al. (2021), in their systematic review, claimed 30-day MALE rates ranging from 0% to 50% in patients receiving either surgical or endovascular treatments, there was no pooled effect recorded in the meta-analyses [12]. PROMISE II was the only study with MACEs reported in 15 of 105 patients over the follow-up period [10].

Yunir et al. (2022) demonstrated diabetes mellitus and smoking to be associated with increased mortality in CLTI patients following endovascular revascularization [43]. A recent cohort study by Kotov et al. (2022) reported inferior outcomes following arterial revascularization in these patients with concomitant CKD, including a significantly higher risk of mortality, major amputations and MACEs in 1 year [44]. Moreover, the PROMISE II trial identified dialysis as the sole risk factor associated with mortality in CLTI patients undergoing pDVA [10]. In our univariate meta-regression analysis, none of the patients’ comorbidities significantly impacted technical success, the efficacy of pDVA or mortality. The role of dialysis could not be adequately investigated in this study due to insufficient data. Although these results do not reflect the impact of patients’ comorbidities on postinterventional outcomes as reported in the literature, they should be interpreted cautiously, as the number of included studies was too low.

### Limitations

This review and meta-analysis is subject to several limitations and the conclusions should be interpreted in consideration of these limitations. Only a small number of studies met the inclusion criteria, thereby limiting the generalizability of the findings. Additionally, most studies had small sample sizes, which might have influenced the statistical power of the analyses. Furthermore, the study populations might vary across the studies with respect to the definition of no-option CLTI, as the level of expertise in endovascular techniques might vary across different centers. To minimize the heterogeneity of the treated patients, one study that assessed those with Rutherford class 4 was excluded [34]. Furthermore, all the included studies were observational. Although our results were statistically significant, the observational nature of the included studies may downgrade the strength of evidence, as large-scale uncontested results from meta-analyses of observational studies are quite uncommon [45]. Therefore, the results may be interpreted cautiously in the setting of clinical decision-making. Additionally, all included studies were single-arm with no comparison groups. However, as there is still no standard therapy for no-option CLTI, it would not be possible to correlate the results with a comparable control group. While the quality of the included studies was generally good, three studies were retrospective in nature, potentially introducing recall bias. Regarding patency rates, the available data were insufficient for meaningful analysis. This limitation highlights the need for further studies with larger and more comprehensive datasets. Finally, the inclusion of papers only from the English-language literature might have been a source of language bias, potentially excluding relevant studies conducted in other languages.

## 5. Conclusions

Taken together, the current systematic review and meta-analysis depicts percutaneous deep vein arterialization not only to be a technically feasible and effective procedure but also a potential limb-rescue strategy in patients with no-option CLTI. Furthermore, the limb salvage and amputation-free survival rates observed at 30 days, 6 months and 1 year imply that the first 6 months of the follow-up serve as the critical window, even though a high rate of short-term reinterventions may be warranted to achieve complete wound healing and limb salvage. Finally, the sparsity of data alongside the single-arm observational nature of the available studies calls for future well-designed controlled trials to investigate the short- and long-term efficacy and safety of pDVA.

## Figures and Tables

**Table 1:** Characteristics of the included studies.

Author	Year	Country	Study Design	No. of Patients	Follow-Up ± SD (Months)	Mean Age ± SD (Years)	Rutherford Class 5/6	Outcomes
Shishehbor et al. [10]	2023	United States	Prospective observational	105	6 ± 0	69.29 ± 10.13	68/37	Technical and procedural success, limb salvage at 30 days and 6 months, overall survival and AFS at 6 months, MALEs, wound healing, reintervention
Miranda et al. [15]	2022	United States	Prospective observational	21	7.13 ± 2.32	68.14 ± 5.29	12/9	Technical success; limb salvage at 6 months and 1 year; overall survival at 30 days, 6 months and 1 year; MALEs; wound healing; reintervention
Clair et al. [22]	2021	United States	Prospective observational	32	13.9 ± 10.05	71 ± 14	28/4	Technical and procedural success; limb salvage, overall survival and AFS at 30 days, 6 months and 1 year; wound healing; reintervention
Nakama et al. [11]	2021	Japan	Retrospective observational	18	7.94 ± 4.84	75.5 ± 8.5	6/12	Technical and procedural success; limb salvage, overall survival and AFS at 30 days, 6 months and 1 year; MALEs; wound healing
Schmidt et al. [14]	2020	The Netherlands, Germany, France, Singapore	Retrospective observational	32	35.26 ± 11.37	67 ± 14	23/9	Technical success, limb salvage, overall survival and AFS at 30 days, 6 months and 1 year; wound healing; reintervention

**Table 2:** Baseline characteristics of study population.

Patient Characteristics	No. of Studies	No. of Patients (%)
Male gender	5	143 (69)
History of smoking	4	93 (45)
Diabetes mellitus	5	156 (75)
Hypertension	5	186 (89)
Chronic kidney disease	5	97 (47)
Rutherford classification	5	
5		137 (66)
6		71 (34)
WIfI * score	4	
0		0 (0)
1		10 (5)
2		25 (12)
3		67 (32)

* WIfI: Wound, Ischemia, foot Infection.

**Table 3:** Details of risk of bias assessment using MINORS criteria.

	Shishehbor, 2023 [10]	Miranda, 2022 [15]	Clair, 2021 [22]	Nakama, 2021 [11]	Schmidt, 2020 [14]
1. A clearly stated aim	2	2	2	2	2
2. Inclusion of consecutive patients	2	2	2	2	2
3. Prospective collection of data	2	2	2	1	1
4. Endpoints appropriate to the aim of the study	2	2	2	2	2
5. Unbiased assessment of the study endpoint	0	0	0	0	0
6. Follow-up period appropriate to the aim of the study	2	2	2	2	2
7. Loss to follow up less than 5%	2	0	1	2	2
8. Prospective calculation of the study size	1	1	1	1	1
9. Additional criteria in the case of comparative study					
10. An adequate control group	NA	NA	NA	NA	NA
11. Contemporary groups	NA	NA	NA	NA	NA
12. Baseline equivalence of groups	NA	NA	NA	NA	NA
13. Adequate statistical analyses	NA	NA	NA	NA	NA
MINORS total score	13	11	12	12	12

**Table 4:** Pooled rates (95% confidence intervals (CIs)) for secondary outcomes after pDVA.

Outcome	No. of Estimates	Summary Rate (95% CI)	Z-Value	$\mathrm{Between}\;\mathrm{Studies}\;I^{2}$ $\;(P_{heterogenesity})$
Procedural success	3	0.740 (0.665–0.803)	5.666	0.00% (0.409)
30-day overall survival	4	0.961 (0.894–0.986)	5.868	0.00% (0.763)
6-month overall survival	5	0.900 (0.850–0.934)	9.360	0.00% (0.872)
1-year overall survival	4	0.851 (0.765–0.909)	6.098	0.00% (0.543)
30-day limb salvage	4	0.904 (0.850–0.941)	8.551	34.57% (0.205)
6-month limb salvage	5	0.771 (0.708–0.824)	7.238	0.00% (0.508)
1-year limb salvage	4	0.767 (0.674–0.840)	5.026	0.00% (0.633)
30-day amputation-free survival	3	0.878 (0.780–0.936)	5.453	31.76% (0.231)
6-month amputation-free survival	4	0.687 (0.616–0.751)	4.884	44.20% (0.146)
1-year amputation-free survival	3	0.656 (0.545–0.752)	2.722	25.97% (0.259)
Primary patency	2	0.308 (0.218–0.397)	6.714	0.00% (0.360)
Primary assisted patency *	1	–	–	–
Secondary patency *	1	–	–	–
Change in Rutherford class *	1	–	–	–
MALE during follow-up	3	0.537 (0.373–0.700)	6.423	0.00% (0.760)
MACE during follow-up *	1	–	–	–
Complete wound healing during follow-up	5	0.534 (0.303–0.765)	4.529	78.53% (0.001)
Reintervention during follow-up	4	0.467 (0.371–0.563)	9.568	45.95% (0.136)

* Too few studies for analysis.

## Supplementary Materials

The following supporting information can be downloaded at: https://www.mdpi.com/article/10.3390/biomedicines12020318/s1.
