# Supplementary material for: Short-Term Outcomes and Efficacy of Percutaneous Deep Vein Arterialization for No-Option Critical Limb Ischemia: A Systematic Review and Meta-Analysis
Source: Biomedicines. 2024 Jan 30;12(2):318. doi: 10.3390/biomedicines12020318 (PMC10886738; doi:10.3390/biomedicines12020318)
Supplement: Supplementary file 1 [file biomedicines-12-00318-s001.zip › biomedicines-2809284-supplementary.pdf]

Table of Contents

Supplemental Table S1. Detailed search strategy for systematic review……………………………………. 2

Supplemental Table S2. List of excluded studies and the reason for exclusion……………………………... 4

Supplemental Table S1. Complete search strategy.

PubMed<br>*June 29, 2023*	
**Query**	**Items found**
((((((((((Percutaneous Deep Venous Arterialization) OR (Percutaneous Deep Vein Arterialization)) OR (Deep Calf Veins Arterialization)) OR (Transcatheter Deep Vein Arterialization)) OR (Transcatheter Deep Venous Arterialization)) OR (Percutaneous Vein Arterialization)) OR (Transcatheter Vein Arterialization)) OR (Endovascular Vein Arterialization)) OR (Endovascular Deep Venous Arterialization)) OR (Endovascular Deep Vein Arterialization)) OR (Endovascular Deep Calf Veins Arterialization)) AND ((("Peripheral Arterial Disease"[Mesh:NoExp]) OR ((((((((((Arterial Disease, Peripheral) OR (Arterial Diseases, Peripheral)) OR (Disease, Peripheral Arterial)) OR (Diseases, Peripheral Arterial)) OR (Peripheral Arterial Diseases)) OR (Peripheral Artery Disease)) OR (Artery Disease, Peripheral)) OR (Artery Diseases, Peripheral)) OR (Disease, Peripheral Artery)) OR (Diseases, Peripheral Artery)) OR (Peripheral Artery Diseases))) OR (((((((Chronic Limb Threatening Ischemia) OR (Ischemia, Chronic Limb-Threatening)) OR (Limb-Threatening Ischemia, Chronic)) OR (Critical Limb Ischemia)) OR (Ischemia, Critical Limb)) OR (Limb Ischemia, Critical)) OR ((No-option Chronic Limb-Threatening Ischemia) OR (No-option Critical Limb-Threatening Ischemia) OR (No-option Chronic Limb Threatening Ischemia) OR (No-option Critical Limb Threatening Ischemia) AND ((humans[Filter]) AND (english[Filter]))) AND ((humans[Filter]) AND (english[Filter]))) AND ((humans[Filter]) AND (english[Filter]))) Filters: Humans, English	843

CENTRAL<br>*June 29, 2023*			
**Query**			**Items found**
ID	Search	Hits	
#1	MeSH descriptor: [Peripheral Arterial Disease] explode all trees	2980	
#2	("peripheral arterial disease"):ti,ab,kw OR ("peripheral arterial occlusive disease"):ti,ab,kw OR ("peripheral arterial obstructive disease"):ti,ab,kw OR ("peripheral arterial occlusion"):ti,ab,kw	3724	
#3	#1 OR #2	4408	
#4	MeSH descriptor: [Chronic Limb-Threatening Ischemia] explode all trees	19	
#5	("critical limb ischæmias"):ti,ab,kw OR ("critical limb ischæmia"):ti,ab,kw OR ("critical limb ischaemia"):ti,ab,kw OR ("critical limb ischaemias"):ti, ab,kw OR ("critical limb ischemia"):ti,ab,kw	849	2
#6	#4 OR #5	855	
#7	(no-option critical limb ischemia):ti,ab,kw OR (no-option chronic limb ischemia):ti,ab,kw	47	
#8	#6 OR #7	859	
#9	(Percutaneous Deep Venous Arterialization):ti,ab,kw OR (Percutaneous Deep Vein Arterialization):ti,ab,kw OR (Deep Calf Veins Arterialization) :ti,ab,kw OR (Transcatheter Deep Vein Arterialization):ti,ab,kw OR		

	(Transcatheter Deep Venous Arterialization):ti,ab,kw	1
#10	(Percutaneous Vein Arterialization):ti,ab,kw OR (Transcatheter Vein Arterialization):ti,ab,kw OR (Endovascular Vein Arterialization):ti,ab,kw OR (Endovascular Deep Venous Arterialization):ti,ab,kw OR (Endovascular Deep Vein Arterialization):ti,ab,kw	1
#11	vein arterialization):ti,ab,kw OR (deep vein arterialization):ti,ab,kw OR (deep venous arterialization):ti,ab,kw	18
#12	#9 OR #10 OR #11	18
#13	#8 AND #12	2

Embase
June 29, 2023

Query	Items found
(('chronic limb threatening ischemia':ti,ab,kw OR 'ischemia, chronic limb-threatening':ti,ab,kw OR 'limb-threatening ischemia, chronic':ti,ab,kw OR 'critical limb ischemia':ti,ab,kw OR 'ischemia, critical limb':ti,ab,kw OR 'limb ischemia, critical':ti,ab,kw) OR ('no-option chronic limb-threatening ischemia':ti,ab,kw OR 'no-option critical limb-threatening ischemia':ti,ab,kw OR 'no-option chronic limb threatening ischemia':ti,ab,kw OR 'no-option critical limb threatening ischemia':ti,ab,kw) OR ('peripheral arterial disease':ti,ab,kw OR 'peripheral artery disease':ti,ab,kw OR 'artery disease, peripheral':ti,ab,kw OR 'artery diseases, peripheral':ti,ab,kw OR 'disease, peripheral artery':ti,ab,kw OR 'diseases, peripheral artery':ti,ab,kw OR 'peripheral artery diseases':ti,ab,kw)) AND ('peripheral arterial disease':ti,ab,kw OR 'peripheral artery disease':ti,ab,kw OR 'artery disease, peripheral':ti,ab,kw OR 'artery diseases, peripheral':ti,ab,kw OR 'disease, peripheral artery':ti,ab,kw OR 'diseases, peripheral artery':ti,ab,kw OR 'peripheral artery diseases':ti,ab,kw)	41

Web of Science
June 29, 2023

Query	Items found
((((((((((((TS=(Peripheral Arterial Disease)) OR TS=(Arterial Disease, Peripheral)) OR TS=(Arterial Diseases, Peripheral)) OR TS=(Disease, Peripheral Arterial)) OR TS=(Diseases, Peripheral Arterial)) OR TS=(Peripheral Arterial Diseases)) OR TS=(Peripheral Artery Disease)) OR TS=(Artery Disease, Peripheral)) OR TS=(Artery Diseases, Peripheral)) OR TS=(Disease, Peripheral Artery)) OR TS=(Diseases, Peripheral Artery)) OR TS=(Peripheral Artery Diseases)) OR ((((((((((TS=(Chronic Limb-Threatening Ischemia)) OR TS=(Chronic Limb Threatening Ischemia)) OR TS=(Ischemia, Chronic Limb-Threatening)) OR TS=(Limb-Threatening Ischemia, Chronic)) OR TS=(Critical Limb Ischemia)) OR TS=(Ischemia, Critical Limb)) OR TS=(Limb Ischemia, Critical)) OR TS=(No-option Chronic Limb-Threatening Ischemia)) OR TS=(No-option Critical Limb-Threatening Ischemia)) OR TS=(No-option Chronic Limb Threatening Ischemia)) OR TS=(No-option Critical Limb Threatening Ischemia))) AND ((((((((((TS=(Percutaneous Deep Venous Arterialization)) OR TS=(Percutaneous Deep Vein Arterialization)) OR TS=(Deep Calf Veins Arterialization)) OR TS=(Transcatheter Deep Vein Arterialization)) OR TS=(Transcatheter Deep Venous Arterialization)) OR TS=(Percutaneous Vein Arterialization)) OR TS=(Transcatheter Vein Arterialization)) OR TS=(Endovascular Vein Arterialization)) OR TS=(Endovascular Deep Venous Arterialization)) OR TS=(Endovascular Deep Vein Arterialization)) OR TS=(Endovascular Deep Calf Veins Arterialization))	56

Supplemental Table S2. List of the excluded studies.

	Publication	Reason for Exclusion
1	Alexandrescu V, Ngongang C, Vincent G, Ledent G, Hubermont G. Deep calf veins arterialization for inferior limb preservation in diabetic patients with extended ischaemic wounds, unfit for direct arterial reconstruction: preliminary results according to an angiosome model of perfusion. Cardiovasc Revasc Med. 2011 Jan-Feb;12(1):10-9. doi: 10.1016/j.carrev.2009.12.002. Epub 2010 Oct 20. PMID: 21241966.	Irrelevant intervention
2	Schreve MA, Minnee RC, Bosma J, Leijdekkers VJ, Idu MM, Vahl AC. Comparative study of venous arterialization and pedal bypass in a patient cohort with critical limb ischemia. Ann Vasc Surg. 2014 Jul;28(5):1123-7. doi: 10.1016/j.avsg.2013.08.010. Epub 2013 Nov 1. PMID: 24189192.	Irrelevant intervention
3	Schreve MA, Lichtenberg M, Ünlü Ç, Branzan D, Schmidt A, van den Heuvel DAF, Blessing E, Brodmann M, Cabane V, Lin WTQ, Kum S. PROMISE international; a clinical post marketing trial investigating the percutaneous deep vein arterialization (LimFlow) in the treatment of no-option chronic limb ischemia patient. CVIR Endovasc. 2019 Jul 31;2(1):26. doi: 10.1186/s42155-019-0067-z. PMID: 32026120; PMCID: PMC6966404.	Study protocol
4	Mutirangura P, Ruangsetakit C, Wongwanit C, Sermsathanasawadi N, Chinsakchai K. Pedal bypass with deep venous arterialization: the therapeutic option in critical limb ischemia and unreconstructable distal arteries. Vascular. 2011 Dec;19(6):313-9. doi: 10.1258/vasc.2010.oa0278. Epub 2011 Oct 18. PMID: 22008976.	Irrelevant intervention
5	Huizing E, Schreve MA, Kum S, Papageorgiou G, de Vries JPM, de Borst GJ, Ünlü Ç. Development of a Prediction Model for the Occurrence of Stenosis or Occlusion after Percutaneous Deep Venous Arterialization. Diagnostics (Basel). 2021 May 31;11(6):1008. doi: 10.3390/diagnostics11061008. PMID: 34073045; PMCID: PMC8226684.	No report of outcome of interest
6	Djoric P, Zeleskov-Djoric J, Stanisavljevic DM, Markovic ZD, Zivkovic V, Vuletic M, Djuric D, Jakovljevic V. Distal venous arterialization and reperfusion injury: focus on oxidative status. Eur Surg Res. 2012;48(4):200-7. doi: 10.1159/000338619. Epub 2012 Jun 7. PMID: 22678100.	Irrelevant intervention
7	Satam K, Aboian E, Huttler J, Zhuo H, Zhang Y, Tonnessen B, Cardella J, Guzman RJ, Ochoa Chaar CI. Eligibility of Patients with Chronic Limb Threatening Ischemia for Deep Venous Arterialization. Ann Vasc Surg. 2022 Oct;86:260-267. doi: 10.1016/j.avsg.2022.04.051. Epub 2022 May 16. PMID: 35589034.	No report of outcome of interest
8	Ferraresi R, Casini A, Losurdo F, Caminiti M, Ucci A, Longhi M, Schreve M, Lichtenberg M, Kum S, Clerici G. Hybrid Foot Vein Arterialization in No-Option Patients With Critical Limb Ischemia: A Preliminary Report. J Endovasc Ther. 2019 Feb;26(1):7-17. doi: 10.1177/1526602818820792. Epub 2018 Dec 28. PMID: 30591004.	Irrelevant intervention
9	Taylor RS, Belli AM, Jacob S. Distal venous arterialisation for salvage of critically ischaemic inoperable limbs. Lancet. 1999 Dec 4;354(9194):1962-5. doi: 10.1016/s0140-6736(99)03164-5. PMID: 10622299.	Irrelevant intervention
10	Engelke C, Morgan RA, Quarmby JW, Taylor RS, Belli AM. Distal venous arterialization for lower limb salvage: angiographic appearances and interventional procedures. Radiographics. 2001 Sep-Oct;21(5):1239-48; discussion 1248-50. doi: 10.1148/radiographics.21.5.g01se281239. PMID: 11553829.	Irrelevant intervention
11	Schreve MA, Huizing E, Kum S, de Vries JPM, de Borst GJ, Ünlü Ç. Volume Flow and Peak Systolic Velocity of the Arteriovenous Circuit in Patients after Percutaneous Deep Venous Arterialization. Diagnostics (Basel). 2020 Sep	Overlap with Schmidt et al. (2020)

	28;10(10):760. doi: 10.3390/diagnostics10100760. PMID: 32998196; PMCID: PMC7601062.	
12	Saab FA, Mustapha JA, Ansari M, Pupp G, Madassery K, N'Dandu Z, et al. Percutaneous Deep Venous Arterialization: Treatment of Patients with End-Stage Plantar Disease. Journal of the Society for Cardiovascular Angiography & Interventions [Internet]. 2022 Nov 1;1(6). Available from: https://doi.org/10.1016/j.jscai.2022.100437	Combination with Rutherford class 4
